# Comparative Functional Analysis of Wheat (*Triticum aestivum*) Zinc Finger-Containing Glycine-Rich RNA-Binding Proteins in Response to Abiotic Stresses

**DOI:** 10.1371/journal.pone.0096877

**Published:** 2014-05-06

**Authors:** Tao Xu, Lili Gu, Min Ji Choi, Ryeo Jin Kim, Mi Chung Suh, Hunseung Kang

**Affiliations:** 1 Department of Plant Biotechnology, College of Agriculture and Life Sciences, Chonnam National University, Gwangju, Korea; 2 Department of Bioenergy Science and Technology, College of Agriculture and Life Sciences, Chonnam National University, Gwangju, Korea; RIKEN Center for Sustainable Resource Science, Japan

## Abstract

Although the functional roles of zinc finger-containing glycine-rich RNA-binding proteins (RZs) have been characterized in several plant species, including *Arabidopsis thaliana* and rice (*Oryza sativa*), the physiological functions of RZs in wheat (*Triticum aestivum*) remain largely unknown. Here, the functional roles of the three wheat RZ family members, named TaRZ1, TaRZ2, and TaRZ3, were investigated using transgenic *Arabidopsis* plants under various abiotic stress conditions. Expression of *TaRZs* was markedly regulated by salt, dehydration, or cold stress. The TaRZ1 and TaRZ3 proteins were localized to the nucleus, whereas the TaRZ2 protein was localized to the nucleus, endoplasmic reticulum, and cytoplasm. Germination of all three TaRZ-expressing transgenic *Arabidopsis* seeds was retarded compared with that of wild-type seeds under salt stress conditions, whereas germination of TaRZ2- or TaRZ3-expressing transgenic *Arabidopsis* seeds was retarded under dehydration stress conditions. Seedling growth of TaRZ1-expressing transgenic plants was severely inhibited under cold or salt stress conditions, and seedling growth of TaRZ2-expressing plants was inhibited under salt stress conditions. By contrast, expression of TaRZ3 did not affect seedling growth of transgenic plants under any of the stress conditions. In addition, expression of TaRZ2 conferred freeze tolerance in *Arabidopsis*. Taken together, these results suggest that different TaRZ family members play various roles in seed germination, seedling growth, and freeze tolerance in plants under abiotic stress.

## Introduction

Gene expression at the posttranscriptional level, including RNA processing, splicing, transport, decay, and translation control, is regulated directly or indirectly by a variety of RNA-binding protein (RBPs) [1], [2]. Typical RBPs contain one or two RNA-recognition motifs (RRMs) at their N-terminus and various auxiliary motifs or regions at their C-terminus, such as glycine-rich region, arginine-rich motif, RGG box, zinc finger motif, and SR- or RD-repeat regions [3]. According to recent progress in genome sequencing of plants, many RBPs have been identified in different plant species, including *Arabidopsis thaliana*, rice (*Oryza sativa*), wheat (*Triticum aestivum*), tobacco, rapeseed, and *Camelina*. It has been demonstrated that these RBPs are involved in genome organization, plant development, and stress responses [4]–[6].

Glycine-rich RNA-binding proteins (GRPs) are characterized by the presence of 1–3 RRMs at the N-terminus and a glycine-rich region at the C-terminus, which play a prominent role in plant responses to diverse abiotic and biotic stresses [4]–[6]. Among the different GRP family members, one GRP family, designated RZs, harbors a CCHC-type zinc finger motif between the N-terminal RRM domain and the C-terminal glycine-rich region [3], [7]. The functional roles of RZs in plant stress response and adaptation have been demonstrated in *Arabidopsis* and rice [8]–[11]. *Arabidopsis AtRZ-1a* is strongly up-regulated by cold stress and down-regulated by dehydration stress or exogenous abscisic acid [8], and affects seed germination and seedling growth of plants under salt, dehydration, or cold stress [8], [11], [12]. In comparison, neither loss-of-function nor overexpression of AtRZ-1b or AtRZ-1c affects seed germination and seedling growth of plants under stress [9]. The rice genome encodes three RZ genes, named OsRZ1, OsRZ2, and OsRZ3. The transcript levels of all three *OsRZs* are up-regulated by cold stress, but are not significantly affected by drought or high salt stress [10]. OsRZ2, but not OsRZ1 and OsRZ3, possess RNA chaperone activity and rescue the cold-sensitive *Arabidopsis grp7* mutant from cold and freezing damage [10]. Although all of these previous reports clearly point to the important roles of RZ family members in the response of certain plant species to different stresses, the stress-responsive roles of RZs in other crop plants, including wheat, have yet to be determined.

The wheat genome harbors four genes encoding RZ proteins, named TaRZ1 (AF315811), TaRZ2 (AK335985), TaRZ3 (tplb0013k12), and TaRZ4 (AK331499). The *TaRZ4* is unusual in that it encodes several different open reading frames with initiation and stop codons at different positions. Our previous study showed that TaRZ2, but not TaRZ3, has RNA chaperone activity [13]. In this study, the functional roles of three wheat RZ family members (TaRZ1, TaRZ2, and TaRZ3) were investigated using transgenic *Arabidopsis* plants that express each TaRZ gene under control of the cauliflower mosaic virus 35S promoter. Seed germination, seedling growth, and freeze tolerance of the transgenic plants were compared with each other under various stress conditions, and the functional roles of different TaRZ family members were comparatively analyzed in plants under abiotic stress conditions.

## Results

### Isolation and characterization of TaRZs in wheat

The cDNA sequences of TaRZ1 (AF315811), TaRZ2 (AK335985), and TaTZ3 (tplb0013k12) encode a putative protein of 237, 211, and 293 amino acid residues with a predicted molecular mass of 25.8, 23.0, and 31.9 kDa, respectively (Table 1). All three TaRZs contained well-conserved ribonucleoprotein1 (RNP1) and RNP2 sequences, as well as a C-terminal glycine-rich region, interspersed by a CCHC-type zinc finger (Figure S1A). Sequence alignment showed that TaRZ1 and TaRZ2 have approximately 67% amino acid sequence homology, TaRZ1 and TaRZ3 have approximately 45% amino acid sequence homology, and TaRZ2 and TaRZ3 have approximately 43% amino acid sequence homology (Table 1). Amino acid sequence homology among the three TaRZs, which was calculated using a web-based program (http://www.uniprot.org/align), was approximately 25%. A phylogenetic analysis was conducted to examine the sequence similarity of RZ proteins among wheat, *Arabidopsis*, cabbage (*Brassica rapa*), rice, and maize (*Zea mays*). The results showed that wheat RZ sequences are more homologous to rice and maize RZ sequences than *Arabidopsis* and cabbage RZ sequences (Figure S1B), suggesting that RZ proteins in monocot plants are highly conserved.

**Table 1:** Compilation of TaRZs investigated in this study.

Gene name	Accession no.	Length (aa)	Localization*		Homology (%)+		
					1	2	3
*TaRZ1*	AF315811	237	Nucleus			67	45
*TaRZ2*	AF335985	211	Nucleus				43
*TaRZ3*	tplb0013k12	293	Nucleus/chloroplast				
RT-PCR primers (5′ to 3′)#							
*TaRZ1*	F; ATGTCGGACGCAGATGATTAC			R; ACGATAGTCTCGATCTCCATC			
*TaRZ2*	F; ATGGCTGATGAGGATGAGTAC			R; TCACGGTCACCGTTACGATC			
*TaRZ3*	F; ATGACGGAGAAGGAGGTGGG			R; CAGCTGCACCACGATATCCA			
*Actin*	F; GGTAACATTGTGCTCAGTGGTGG			R; AACGACCTTAATCTTCATGCTGC			

*Cellular localization predicted via PSORT (http://psort.ims.u-tokyo.ac.jp) and TargetP (http://www.cbs.dtu.dk/services/TargetP) programs.

+ Sequence homology (%) predicted via ClustalW program.

# F, forward primer; R, reverse primer.

### Transcript levels, stress-responsive expression patterns, and subcellular localization of TaRZs in wheat

**Figure 1:**
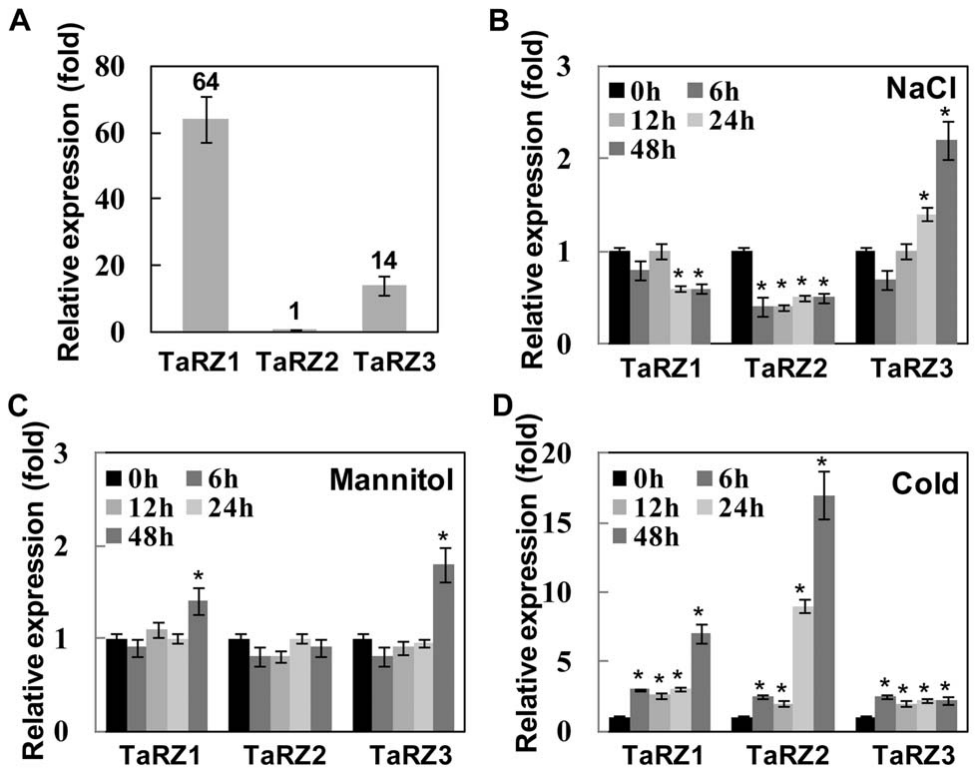
Expression levels and stress-responsive expression patterns of *TaRZs* in wheat. (A) Transcript levels of *TaRZ1*, *TaRZ2*, and *TaRZ3* were analyzed via real-time RT-PCR and presented as relative expression (fold) of *TaRZ2* expression level. Two-week-old wheat plants were subjected to (B) salt, (C) dehydration, or (D) cold stress for 6, 12, 24, and 48 h, and the transcript levels of each *TaRZ* were analyzed via real-time RT-PCR and presented as the relative expression (fold) of the non-stressed controls. Values are means ± SE obtained from five independent experiments. Asterisks above each column indicate values that are statistically different from the control values (p≤0.05).

The transcript levels of three *TaRZs* in the aerial part of 2-week-old wheat seedlings were determined via quantitative real-time RT-PCR analysis. Among the three *TaRZs* in wheat, *TaRZ2* showed the lowest level of expression under normal growth conditions, and the expression levels of *TaRZ*1 and *TaRZ*3 were approximately 64- and 14- fold higher than that of *TaRZ2*, respectively (Figure 1A). The stress-responsive expression patterns of *TaRZs* were then assessed in wheat under salt, dehydration, or cold stress conditions. Wheat plants were subjected to the indicated stress treatments, and the expression levels of *TaRZs* were analyzed by real-time RT-PCR. To compensate for any circadian effect on gene expression, we measured the expression levels of *TaRZs* in non-stressed control plants as well as in stress-treated plants at each time point. Under salt stress conditions, the transcript levels of *TaRZ1* and *TaRZ2* decreased, whereas the transcript levels of *TaRZ3* increased (Figure 1B). Dehydration treatment marginally up-regulated *TaRZ1* and *TaRZ3* transcript levels (Figure 1C). *TaRZ* expression was markedly up-regulated by cold stress as *TaRZ1* and *TaRZ2* transcript levels increased up to 7- or 17- fold, respectively, 48 h after cold treatment, while transcript levels of *TaRZ3* increased 2.5-fold by cold treatment (Figure 1D).

**Figure 2:**
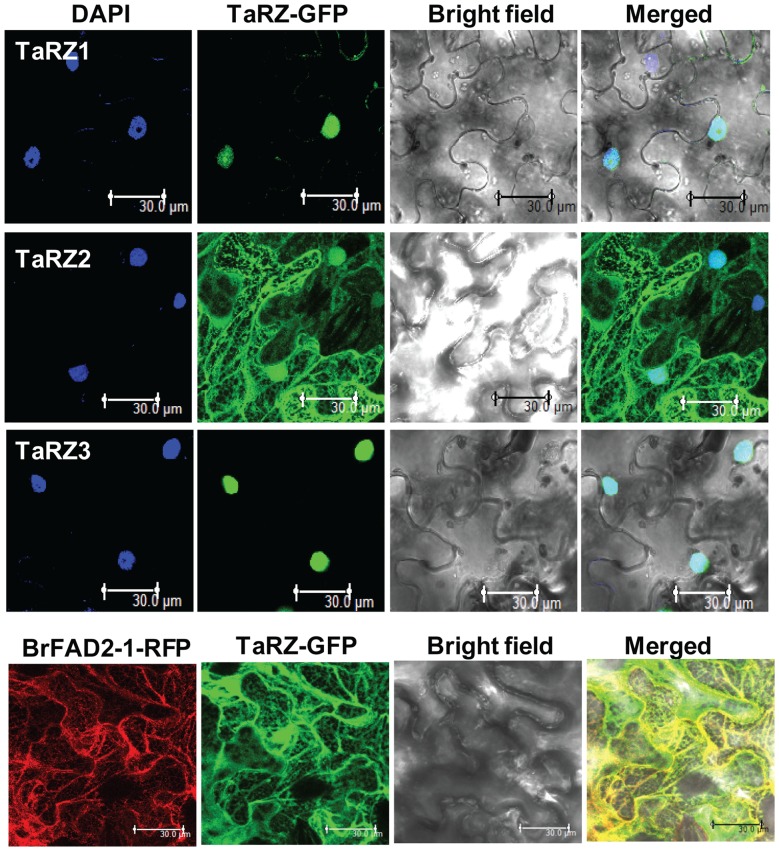
Subcellular localization of TaRZ proteins. TaRZ-GFP fusion proteins were transiently expressed in tobacco plant, and GFP signals in tobacco leaves were detected using a confocal microscope. DAPI was used to stain the nuclei, and *Brassica rapa* microsomal delta-12 fatty acid desaturase (BrFAD2) was used as a marker for ER localization. Bar  = 30 mm.

To determine the subcellular localization of TaRZ proteins, cellular localization was first predicted via the PSORT (http://psort.ims.u-tokyo.ac.jp) and TargetP (http://www.cbs.dtu.dk/services/TargetP) programs. The results showed that TaRZ1 and TaRZ2 proteins were predicted to be localized to the nucleus, and TaRZ3 protein was predicted to be localized mainly to the nucleus and possibly to the chloroplast. To confirm the subcellular localization of TaRZ proteins, TaRZs-green fluorescent protein (GFP) fusion proteins were transiently expression in tobacco under control of the CaMV 35S promoter, and the localization of the fusion proteins was analyzed by confocal microscopy. The results showed that strong GFP signals were detected exclusively in the nucleus of TaRZ1-GFP and TaRZ3-GFP plants (Figure 2). In comparison, GFP signals in TaRZ2-GFP plants were detected in several places, including the nucleus, endoplasmic reticulum (ER), and cytoplasm (Figure 2). Localization of TaRZ2 in the ER was further supported by co-transformation of the tobacco leaves with microsomal delta-12 fatty acid desaturase (FAD2) that is localized to the ER and catalyzes the first committed step of the biosynthesis of polyunsaturated fatty acids [14] (Figure 2). These results indicate that the TaRZ1 and TaRZ3 proteins are localized to the nucleus, whereas the TaRZ2 protein is localized to the nucleus, ER, and cytoplasm.

### TaRZs affect seed germination of *Arabidopsis* under salt or dehydration stress conditions

**Figure 3:**
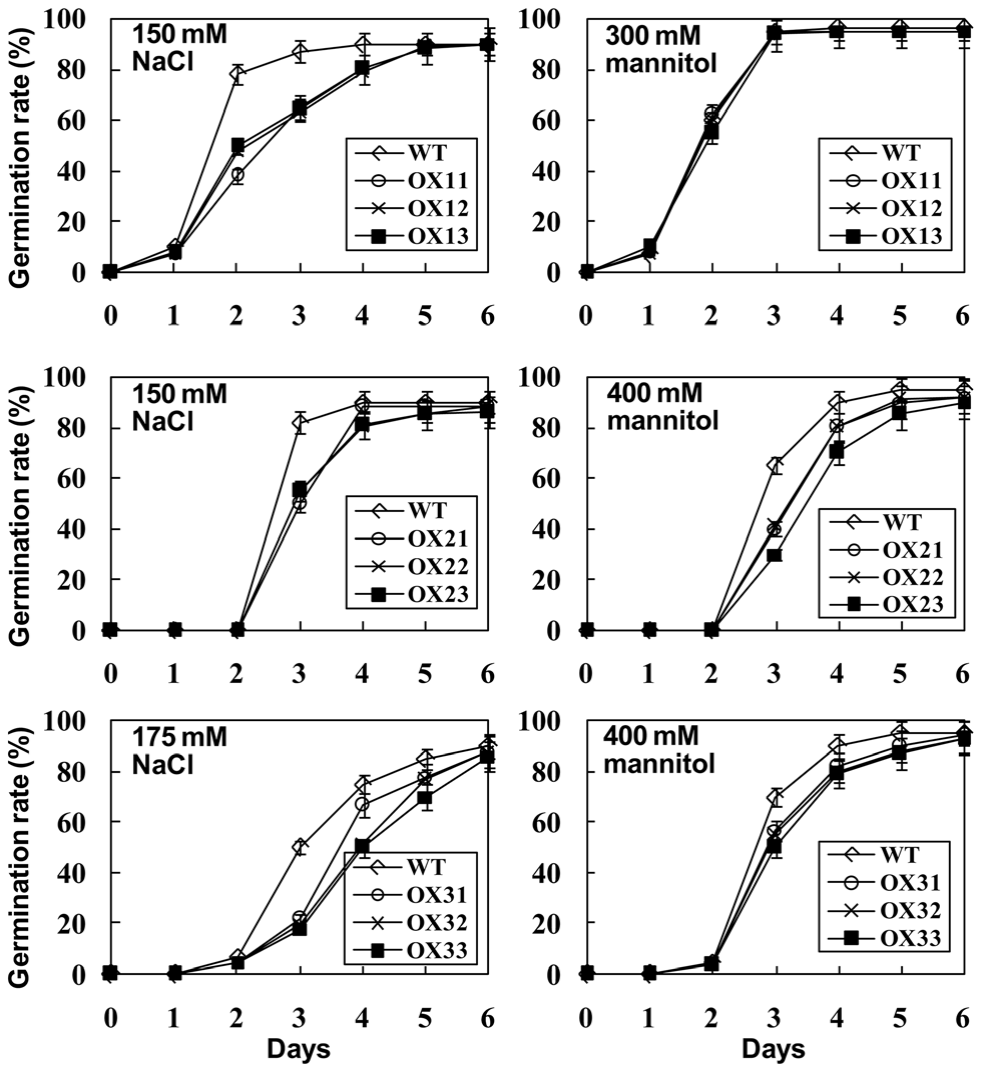
Effect of abiotic stresses on seed germination of transgenic plants. Seeds of the wild-type (WT), TaRZ1-expressing *Arabidopsis* plants (OX11, OX12, and OX13), TaRZ2-expressing *Arabidopsis* plants (OX21, OX22, and OX23), and TaRZ3-expressing *Arabidopsis* plants (OX31, OX32, and OX33) were germinated on MS medium supplemented with NaCl or mannitol at 23°C, and germination rates (%) were scored on the indicated days. Values are means ± SE of three replicates, approximately 100 seeds per replicate.

With the observation that expression levels of *TaRZs* are regulated by different stress conditions, we examined whether TaRZs play any roles in plant stress responses. Because wheat transformation is technically difficult, we investigated TaRZ function using *Arabidopsis*. Multiple transgenic *Arabidopsis* plants expressing each TaRZ under control of the cauliflower mosaic virus 35S promoter were generated, and 3 T_3_ homozygote transgenic lines were selected for phenotypic analysis. Expression of *TaRZs* in each transgenic line was confirmed by RT-PCR analysis (Figure S2). Transgenic *Arabidopsis* plants expressing TaRZ1, TaRZ2, or TaRZ3 under control of the 35S promoter were designated 35S::TaRZ1, 35S::TaRZ2, or 35S::TaRZ3, respectively. To investigate whether TaRZ affects seed germination under various abiotic stress conditions, germination rates of the wild-type and each transgenic seed were evaluated on MS medium containing different concentrations of NaCl or mannitol. Germination rates of the wild-type and transgenic seeds were similar to each other on normal MS medium. However, when the seeds were germinated on MS medium containing NaCl, germination of all transgenic seeds was delayed compared with that of the wild-type seeds (Figure 3). Dehydration stress affected germination of the transgenic seeds differently as germination of 35S::TaRZ2 and 35S::TaRZ3 seeds was delayed compared with that of the wild-type seeds, whereas germination of 35S::TaRZ1 seeds was similar to that of the wild-type seeds on MS medium supplemented with mannitol (Figure 3). When the seeds were germinated at low temperature (10°C), germination rates of all transgenic seeds were similar to that of the wild-type seeds (Figure S3). These results demonstrate that TaRZs affect seed germination differently under salt, dehydration, or cold stress conditions.

### TaRZ1 and TaRZ2 have a negative impact on seedling growth under salt or cold stress conditions

**Figure 4:**
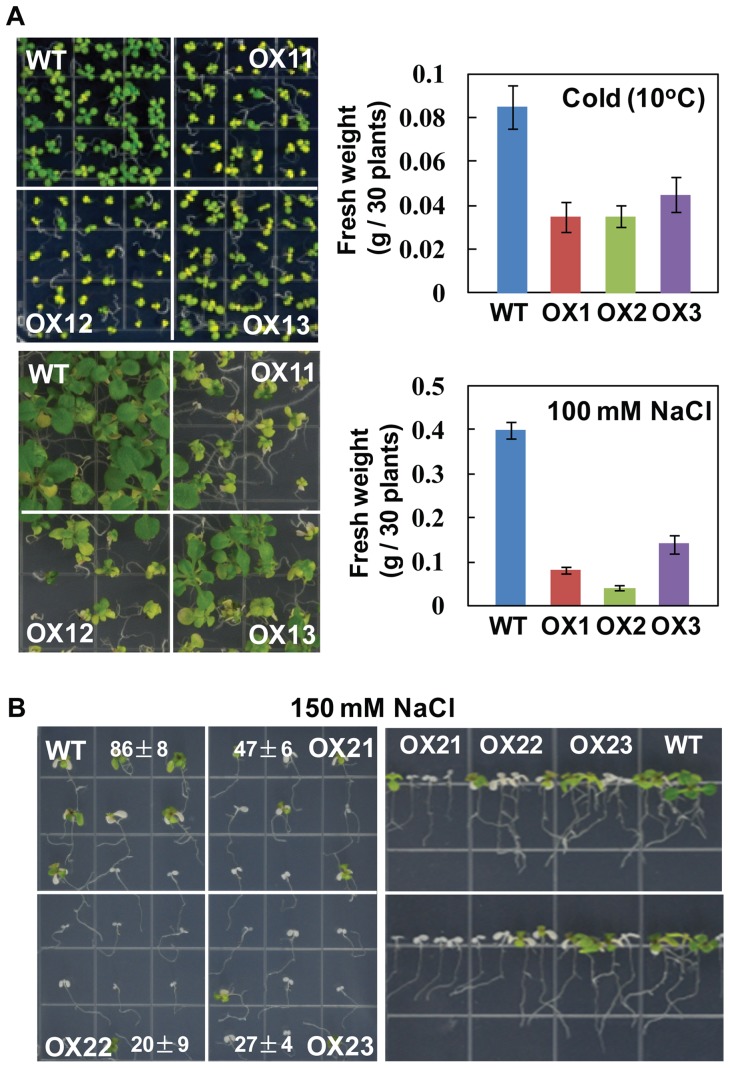
Effect of cold or salt stress on seeding growth of transgenic plants. (A) Seedling growth of the wild-type (WT) and TaRZ1-expressing plants (OX11, OX12, and OX13) was examined on MS medium supplemented with 100 mM NaCl or MS medium at 10°C. Photographs were taken 14 days after stress treatment, and fresh weight (g) of 30 plantlets was measured. (B) Seedling growth of the wild-type (WT) and TaRZ2-expressing plants (OX21, OX22, and OX23) was examined on MS medium supplemented with 150 mM NaCl. Survival rates (%) of seedlings on the left photograph were scored 14 days after germination, and values are means ± SE of five replicates, approximately 10 seedlings per replicate. The right photograph shows leaf greening was markedly inhibited in TaRZ2-expressing seedlings.

As it is evident that TaRZs affected seed germination under stress conditions, we next assessed whether TaRZs play a role in seedling growth under stress conditions. Seeds were first fully germinated on MS medium for 3 days, and the seedlings were transferred to MS medium supplemented with NaCl or mannitol, or the MS plates were placed in a growth chamber maintained at 10°C for stress treatment. No significant differences in seedling and root growth were observed between the wild-type and 35S:: TaRZ3 plants under all stress conditions (Figure S4). By contrast, seedling growth of 35S:: TaRZ1 plants was significantly inhibited under cold or salt stress conditions (Figure 4A), and seedling growth of 35S:: TaRZ2 plants was also inhibited by salt stress (Figure 4B). Dehydration stress did not affect seedling growth of 35S:: TaRZ1 plants (Figure S5A), and dehydration or cold stress had no effect on seedling growth of 35S::TaRZ2 plants (Figure S5B). These differences in seedling growth among the genotypes were consistently observed when tested on MS medium supplemented with different concentrations of NaCl or mannitol. These results demonstrate that TaRZ1 and TaRZ2 negatively affected seedling growth under salt stress conditions and that TaRZ1 negatively influenced seedling growth under cold stress conditions.

### TaRZ2 confers freeze tolerance in *Arabidopsis*

**Figure 5:**
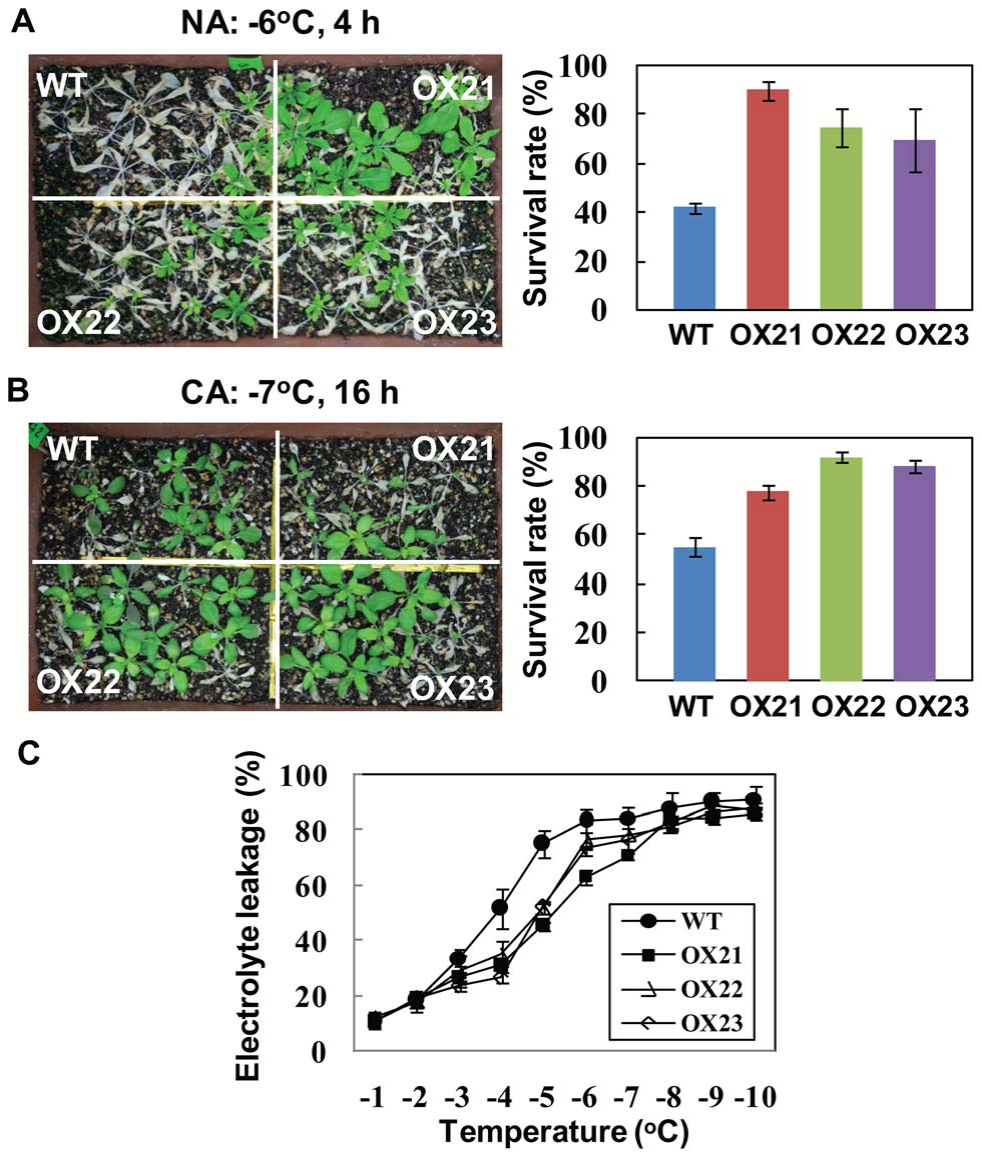
TaRZ2 confers freezing tolerance in *Arabidopsis* plants. (A) For non cold-acclimated (NA) freezing tolerance tests, 4-week-old plants of the wild-type (WT) and TaRZ2-expressing plants (OX21, OX22, and OX23) were subjected to freezing shock at −6 for 3–6 h directly under continuous light, then transferred to normal growth conditions. (B) For cold-acclimated (CA) freezing tolerance test, 4-week-old plants were first placed at 4°C for 1 day, −1°C for 1 day, and then subjected to freezing shock at −7 for 12–25 h. Surviving plants were counted 7 days after transferring to normal growth conditions. One representative picture among repeated experiments was shown. (C) For electrolyte leakage test, leaves from the NA wild-type and TaRZ2-expresing plants were frozen at −1 to −10°C, and the extent of cellular damage was estimated by measuring electrolyte leakage.

Because expression of *TaRZs* was highly up-regulated in wheat under cold stress conditions, TaRZs are likely involved in the response of plants to cold or freezing stress. Although TaRZs did not affect seed germination and seedling growth of *Arabidopsis* under cold stress conditions (Figures S3–S5), we next evaluated the functional role of TaRZs under freezing stress conditions. When the plants were subjected to freezing stress at −6 or −7°C for 4–24 h, survival rates and freeze tolerance of 35S::TaRZ1 and 35S::TaRZ3 plants were similar to those of the wild-type plants. By contrast, survival rates and freeze tolerance of 35S::TaRZ2 plants increased compared with those of the wild-type plants. In non cold-acclimated (NA) freeze tolerance tests, approximately 40% of the wild-type plants survived, whereas 70–80% of 35S::TaRZ2 plants survived after freezing stress at −6°C for 4 h (Figure 5A). In addition, in cold-acclimated (CA) freeze tolerance tests, approximately 55% of the wild-type plants survived, whereas 80–90% of 35S::TaRZ2 plants survived after freezing stress at −7°C for 16 h (Figure 5B). Freeze tolerance of 35S::TaRZ2 plants was consistently observed when CA freezing tests were repeated at −7°C for 12–24 h (Figure S6). To further confirm the role of TaRZ2 in freeze tolerance, the contribution of TaRZ2 to the enhanced freeze tolerance of *Arabidopsis* was evaluated by measuring cellular damage of the plants as a result of freeze-induced membrane lesions. Electrolyte leakage from the leaves of 35S::TaRZ2 plants was much less than that observed in the wild-type when incubated at −1 to −10°C (Figure 5C). All of these results indicate that 35S::TaRZ2 plants are more tolerant to freezing stress than wild type, suggesting that TaRZ2 confers freeze tolerance in *Arabidopsis.*

## Discussion

Our results show that different TaRZ family members play varying roles in seed germination, seedling growth, and freeze tolerance in plants under abiotic stress conditions. Despite the potential role of RZ family members in various plants under stress, the experimental evidences supporting their functional roles are limited to only a few plant species, including *Arabidopsis* and rice. It has been reported that, among the three *Arabidopsis* AtRZs, AtRZ-1a plays a positive role in plant response to cold and freezing stress but negatively affects seed germination and seedling growth of *Arabidopsis* under salt or dehydration stress [8], [11], [12]. By contrast, AtRZ-1b or AtRZ-1c does not affect seed germination and seedling growth of *Arabidopsis* under stress conditions [9]. Here, we demonstrated that wheat TaRZ2 contributes to enhance freeze tolerance of *Arabidopsis* (Figure 5) and plays a negative role during seed germination and seedling growth under salt or dehydration stress conditions (Figure 3 and 4). Sequence analysis shows that TaRZ2 is most homologous to AtRZ1-a among the three AtRZs (Figure S1). Importantly, the cellular localization of TaRZ2 and AtRZ-1a was similar in that they were localized to the nucleus and cytoplasm, which is different from the nucleus localization of other *Arabidopsis* [9] and wheat TaRZs (Figure 2). These present and previous results suggest that AtRZ-1a and TaRZ2 are functionally conserved in *Arabidopsis* and wheat. Interestingly, the up- and down-regulation of *TaRZs* by cold, salt, or dehydration stress was similar to the stress-responsive expression patterns of *AtRZs* [8] and *OsRZs* [10]. We showed previously that, among the three rice OsRZs, only OsRZ2 has the ability to confer cold and freeze tolerance in *Arabidopsis* [10]. All of these results suggest that certain RZ family members are functionally conserved in diverse plant species during the cold and freeze stress response and adaptation.

The next important question is to determine how RZs exert their roles during stress adaptation. Although we do not understand conclusively the mechanistic roles of RZs in the response of plants to all stresses, the function of RZs during cold adaptation could be related to the RNA chaperone activity of RZ proteins. RNA chaperones are non-specific RBPs that interact with diverse RNA substrates and aid RNA folding or structural rearrangement [15], [16]. The function of RNA chaperones is particularly important during cold adaptation in *E. coli*, during which cold shock proteins are highly induced and function as RNA chaperones that destabilize over-stabilized secondary structures in mRNAs, thereby facilitating efficient translation at low temperature [17]–[20]. Notably, among the three TaRZ proteins in different plant species, only TaRZ2, OsRZ2, and AtRZ-1a in wheat, rice, and *Arabidopsis*, respectively, which confer cold and freeze tolerance in plants, harbor RNA chaperone activity [8], [10], [13]. A close link between RNA chaperone activity and cold or freeze tolerance has also been demonstrated in the study of GRP family members. Arabidopsis AtGRP7, which accelerates seed germination and seedling growth under low temperatures and confers freeze tolerance to *Arabidopsis*, harbors RNA chaperone activity [21], [22], whereas AtGRP4, which does not affect seed germination or seedling growth under cold stress conditions [23], harbors no RNA chaperone activity [21]. Importantly, TaRZ2, OsRZ2, and AtGRP7, which harbor RNA chaperone activity, are localized to both the nucleus and cytoplasm (Figure 2) [10], [22]. It is likely that RNA chaperone activity of RZs in the cytoplasm facilitates efficient translation at low temperatures in plants as observed in bacteria [17]–[20]. The mechanistic roles of RZs in plants under salt or dehydration stress conditions are not clearly understood. The expression of *TaRZ1* and *TaRZ2* was down-regulated in wheat by salt stress (Figure 1), and seed germination and seedling growth of TaRZ1- or TaRZ2- expressing plants were retarded under salt stress conditions (Figures 3 and 4). Although the functional role of TaRZ1 and TaRZ2 in wheat under salt stress is not yet understood, these results suggest that down-regulation of *TaRZ1* and *TaRZ2* should be an adaptive response of wheat to salt stress. It is likely that TaRZ1 and TaRZ2 exert their roles by either directly or indirectly controlling the transcript levels and/or RNA metabolism of target mRNAs that are negatively involved in salt stress response in wheat.

In conclusion, our results provide information for a better understanding of the different roles of TaRZ family members under various abiotic stresses. RZ family members in wheat, rice, and *Arabidopsis* are structurally conserved, and some of them are functionally conserved by acting as RNA chaperones under cold or freezing stress conditions. Future efforts are needed to determine functional roles of RZs in wheat under stress conditions. We anticipate that the findings described here will be useful in future studies directed at understanding the functional roles of RZs in the response of wheat to diverse abiotic stresses. Considering that RZ family members should exert their functions by interacting with RNAs, searching for the target RNAs for specific RZs would also be important for a much deeper understanding of the cellular roles and molecular mechanisms of RZs in both monocotyledonous and dicotyledonous plants under stress conditions.

## Materials and Methods

### Plant materials, stress treatments, and expression analysis

The winter wheat used in this study was a *T. aestivum* cv. Keum Kang variety which is cultivated in Korea. Wheat plants were grown in soil at 23°C under 16 h light/8 h dark photocycle. For salt or dehydration stress treatment, 2-week-old wheat seedlings were submerged into a solution containing 300 mM NaCl or 250 mM mannitol, respectively. For cold stress treatment, 2-week-old seedlings were placed in a growth chamber at 4°C. The samples were collected at the indicated time intervals, and total RNA was extracted from the frozen plant samples using the Plant RNeasy extraction kit (Qiagen, Valencia, CA, USA). Transcript levels of each gene were determined via real-time RT-PCR with the gene-specific primers listed in Table 1. All experimental conditions for real-time RT-PCR and data analysis were essentially as described previously [8]. Briefly, real-time quantification of the RNA transcripts was performed in the Rotor-Gene Q thermal cycling system (Qiagen) using QuantiTect SYBR Green RT-PCR kit (Qiagen). The reaction mixture (25 μL) contained 200 ng of total RNA, 0.5 μM of each primer listed in Table 1, and appropriate amounts of enzymes and fluorescent dyes as recommended by the manufacturer (Qiagen). Relative expression levels of each gene were calculated after normalization of the transcript abundance using *Actin* gene as a reference. All experiments were repeated at least three times.

### Vector construction and *Arabidopsis* transformation

To generate transgenic *Arabidopsis* plants expressing each TaRZ, the full-length cDNA of TaRZs was cloned into the BamHI/SalI site of the pCAMBIA 1301 vector, which expresses each TaRZ under control of the CaMV 35S promoter. *Arabidopsis* transformation was performed according to the vacuum infiltration method [24] using *Agrobacterium tumefaciens* GV3101. To identify transgenic plants, seeds were harvested and plated on selection medium containing hygromycin (50 µg ml^−1^) and carbenicillin (250 µg ml^−1^). Expression of each *TaRZ* in the transgenic plants was confirmed by RT-PCR analysis. The T_3_ or T_4_ homozygous lines were selected and used for phenotypic investigation.

### Analysis of cellular localization of TaRZ proteins

To determine cellular localization of TaRZ proteins, the cDNAs corresponding to each TaRZ protein were fused in-frame with GFP. The TaRZ-GFP fusion protein was transiently expressed in tobacco plants under control of the CaMV 35S promoter [25]. Leaf samples were mounted on microscope slides, and the cellular expression of TaRZ-GFP proteins was observed under a confocal microscope (Carl Zeiss, Inc. Thornwood, NY, USA). Excitation and emission wavelengths were 488 and 505–545 nm, respectively. To confirm ER localization of TaRZ2 protein, the tobacco leaves were co-transformed with microsomal delta-12 fatty acid desaturase (FAD2) that is localized to the ER and catalyzes the first committed step of the biosynthesis of polyunsaturated fatty acids [14], and the signals in ER were observed under a confocal microscope.

### Germination and seedling growth assays under abiotic stress conditions

*A. thaliana* (Col-0 ecotype) wild-type and transgenic plants were grown in a growth chamber at 23°C under long day conditions (16 h light/8 h dark). Seed germination and seedling growth under stress conditions were conducted essentially as described previously [22], [26]. Sterilized seeds were sown on half-strength Murashige-Skoog (MS) medium supplemented with 75–175 mM NaCl or 200–300 mM mannitol for salt or dehydration stress treatments, respectively. For low temperature stress treatment, seeds on MS medium were kept in a growth chamber maintained at 10°C. The seeds were regarded as germinated when the radicles protruded from the seed coat. To determine the effect of salt or dehydration stress on seedling growth, the seeds were fully germinated under normal conditions, and 3-day-old seedlings were transferred to MS medium supplemented with NaCl or mannitol. To determine the effect of cold stress on seedling growth, 3-day-old seedlings germinated under normal conditions were placed in a growth chamber maintained at 10°C.

### Freeze tolerance and electrolyte leakage tests

Both non cold-acclimated (NA) and cold-acclimated (CA) freeze tolerance tests were conducted using 21-day-old wild-type and transgenic plants grown in pots. For the NA freeze tolerance tests, the plants were placed at −6°C for 3–6 h directly under continuous light. For the CA freeze tolerance tests, the plates were first placed at 4°C for 1 day and then subjected to a series of temperature treatments; −1°C for1 day, −7°C for 12 h, 16 h and 25 h under continuous light. After freezing shock, the plants were immediately placed at 4°C for 1 day in the dark, and then in a growth chamber under normal conditions. The electrolyte leakage test was conducted essentially as previously described [22]. In brief, the leaves from 3-week-old *Arabidopsis* plants were placed in a test tube containing 100 µl distilled water, and the tube was placed at 0°C for 1 h. An ice crystal was added to the tube, and the temperature of the water bath was decreased to −10°C at a rate of 1°C per 30 min. The tubes were removed from the water bath at the indicated temperatures, and the conductivity of the solution was measured before and after autoclaving for 10 min at 121°C. The ratio of electrolyte content before and after autoclaving was utilized as an indicator for membrane damage. The experiment was repeated at least four times.

## Supporting Information

Figure S1 **Alignment of the amino acid sequences of TaRZs.** (A) The alignment was made using the ClustalW2 program. Gaps in the sequences are indicated by dashes. The positions of ribonucleoprotein1 (RNP1), RNP2, and CCHC zinc finger regions are indicated by thick lines. (B) Relationship of RZ proteins among Arabidopsis, wheat, rice, maize, and cabbage. Phylogenetic tree was generated using the ClustalW2 program (http://www.genome.jp/tools/clustalw/) based on the deduced amino acid sequences of RZ proteins. Accession numbers of RZs are as follow; Arabidopsis (At3g26420, At1g60650, At5g04280), wheat (AF315811, AF335985, tplb0013k12), rice (Os03g61990, Os07g08960, Os03g68190), maize (GRMZM5G874478, GRMZM2G082931, GRMZM2G083783, GRMZM2G161242, GRMZM2G053223), and cabbage (Bra025205, Bra032933, Bra007004, Bra003139, Bra023114, Bra029777).
(TIF)

Figure S2 **Confirmation of overexpression plants.** Transcript levels of TaRZ1- expressing *Arabidopsis* plants (OX11, OX12, and OX13) (A), TaRZ2-expressing *Arabidopsis* plants (OX21, OX22 and OX23) (B), and TaRZ3-expressing *Arabidopsis* plants (OX31, OX32 and OX33) (C) were analyzed by RT-PCR.
(TIF)

Figure S3 **Seed germination of wild-type and transgenic plants under normal conditions.** Seeds of wild type (Col-0) and overexpression plants 35S::TaRZ1 (OX11, OX12 and OX13) and 35S::TaRZ2 (OX21, OX22 and OX23) and 35S::TaRZ3 (OX31, OX32, and OX33) were germinated on MS medium at 24°C, and germination rates were scored at the indicated days.
(TIF)

Figure S4 **Effect of dehydration, cold and salt stress on seeding growth of wild type and 35S::TaRZ3 transgenic plants.** (A) Effect of dehydration on seeding growth and root length of wild type and 35S::TaRZ3 transgenic plants. MS medium supplemented with 300 mM mannitol. (B) Effect of low temperature on seeding growth and root length of wild type and 35S::TaRZ3 transgenic plants. (C) Effect of salt on seeding growth and root length of wild type and 35S::TaRZ3 transgenic plants. MS medium was supplemented with 125 mM NaCl. Values are means ± SD of at least 20 seedlings.
(TIF)

Figure S5 **Effect of dehydration and cold stress on seeding growth of wild type and 35S::TaRZ1 transgenic plants and 35S::TaRZ2 transgenic plants.** (A) Effect of dehydration on seeding growth and root length of wild type and 35S::TaRZ1 transgenic plants. (B) Effect of dehydration and low temperature on seeding growth and root length of wild type and 35S::TaRZ2 transgenic plants. Seedling and root growths of wild type (Col-0) and overexpression plants (OX21 OX22, and OX23) were examined on MS medium supplemented with 300 mM mannitol. Values are means ± SD of at least 30 seedlings.
(TIF)

Figure S6 **Cold-acclimated (CA) freezing tolerance test.** Three-week-old plants were first placed at 4°C for 1 day, −1°C for 1 day, −7°C for 12–25 h, and then recovered under normal growth conditions for 7 d. The photograph shows a representative picture of repeated.
(TIF)
