# Supplementary figures and images for: Comparative Functional Analysis of Wheat (Triticum aestivum) Zinc Finger-Containing Glycine-Rich RNA-Binding Proteins in Response to Abiotic Stresses
Source: PLoS One. 2014 May 6;9(5):e96877. doi: 10.1371/journal.pone.0096877 (PMC4011930; doi:10.1371/journal.pone.0096877)

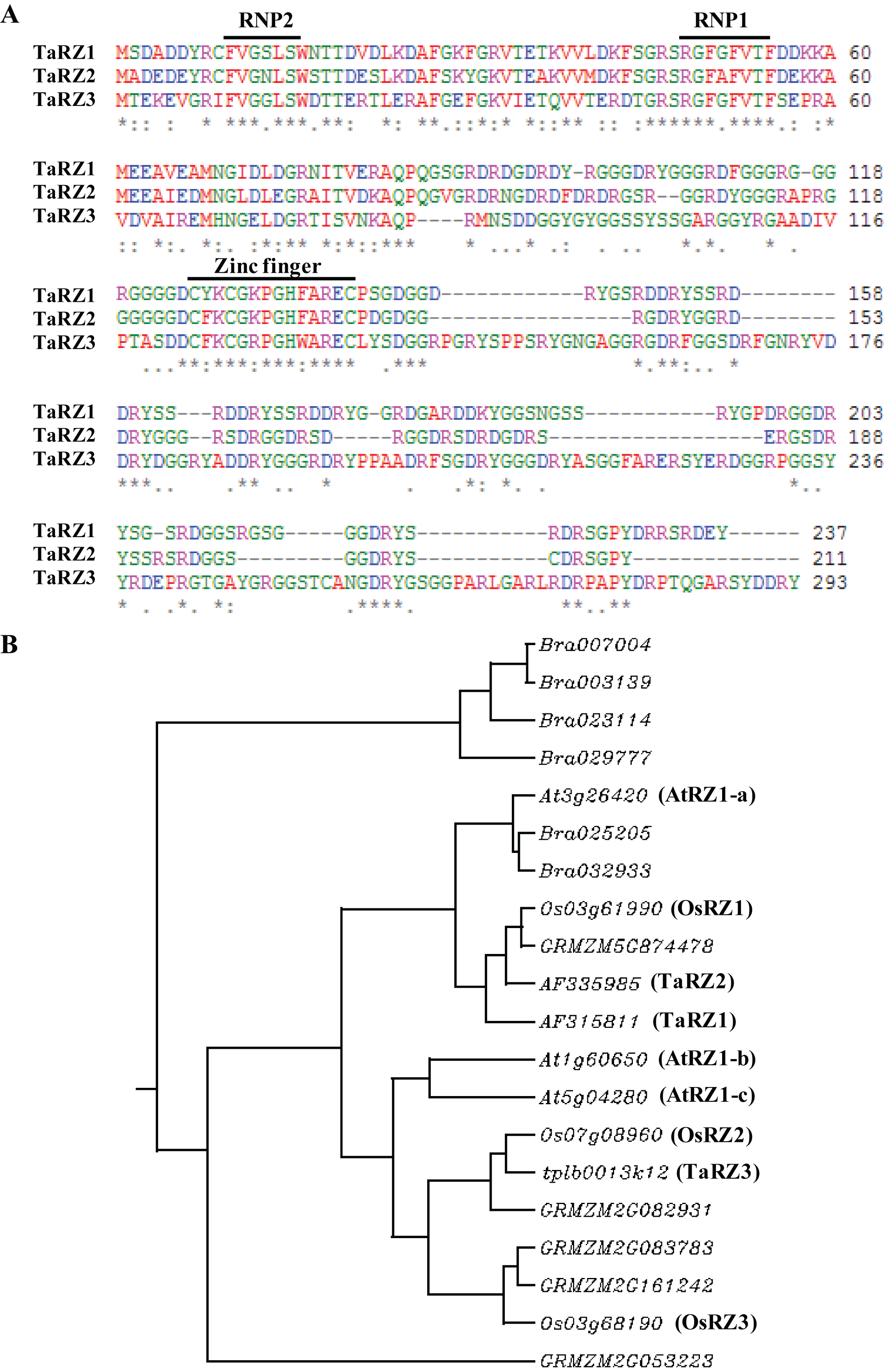

Supplement: Figure S1 — Alignment of the amino acid sequences of TaRZs. (A) The alignment was made using the ClustalW2 program. Gaps in the sequences are indicated by dashes. The positions of ribonucleoprotein1 (RNP1), RNP2, and CCHC zinc finger regions are indicated by thick lines. (B) Relationship of RZ proteins among Arabidopsis, wheat, rice, maize, and cabbage. Phylogenetic tree was generated using the ClustalW2 program (http://www.genome.jp/tools/clustalw/) based on the deduced amino acid sequences of RZ proteins. Accession numbers of RZs are as follow; Arabidopsis (At3g26420, At1g60650, At5g04280), wheat (AF315811, AF335985, tplb0013k12), rice (Os03g61990, Os07g08960, Os03g68190), maize (GRMZM5G874478, GRMZM2G082931, GRMZM2G083783, GRMZM2G161242, GRMZM2G053223), and cabbage (Bra025205, Bra032933, Bra007004, Bra003139, Bra023114, Bra029777). (TIF) [file pone.0096877.s001.tif]

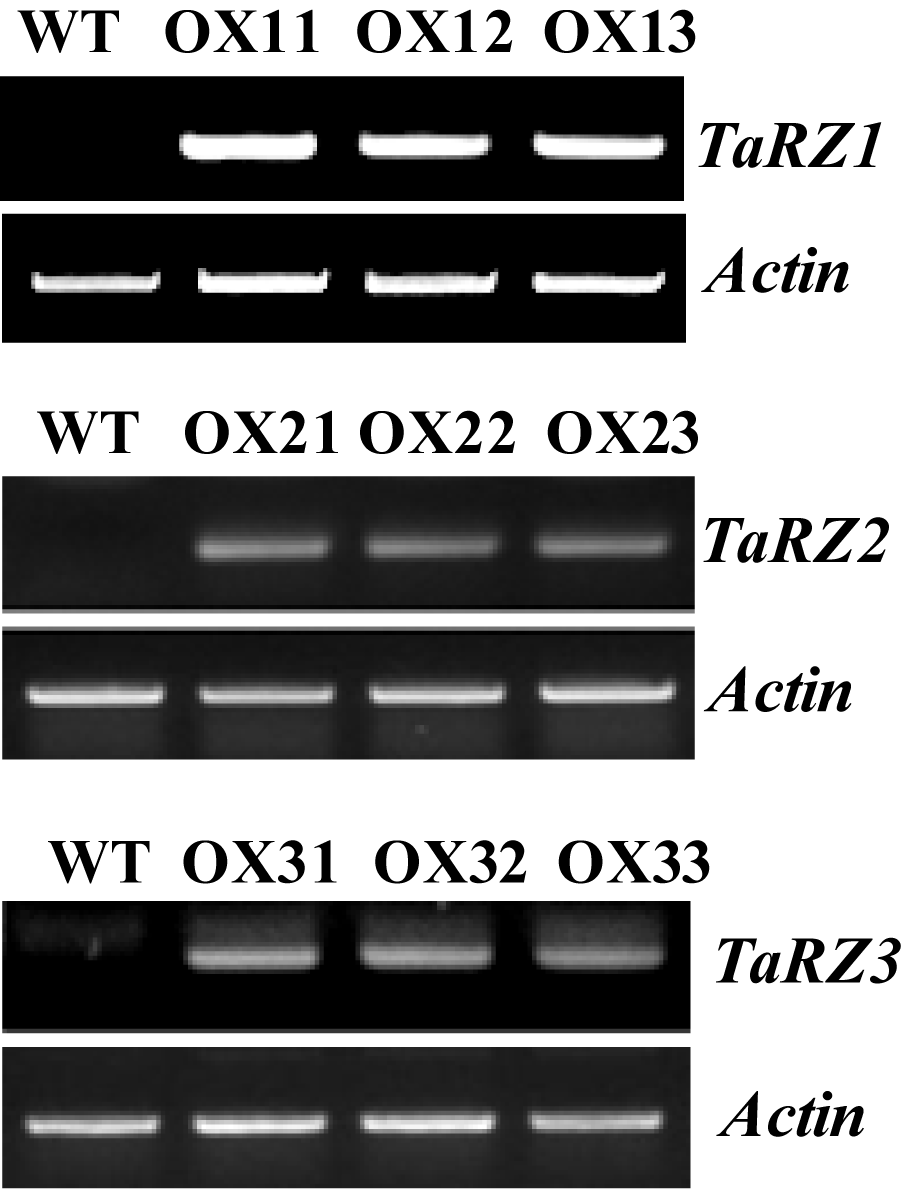

Supplement: Figure S2 — Confirmation of overexpression plants. Transcript levels of TaRZ1- expressing Arabidopsis plants (OX11, OX12, and OX13) (A), TaRZ2-expressing Arabidopsis plants (OX21, OX22 and OX23) (B), and TaRZ3-expressing Arabidopsis plants (OX31, OX32 and OX33) (C) were analyzed by RT-PCR. (TIF) [file pone.0096877.s002.tif]

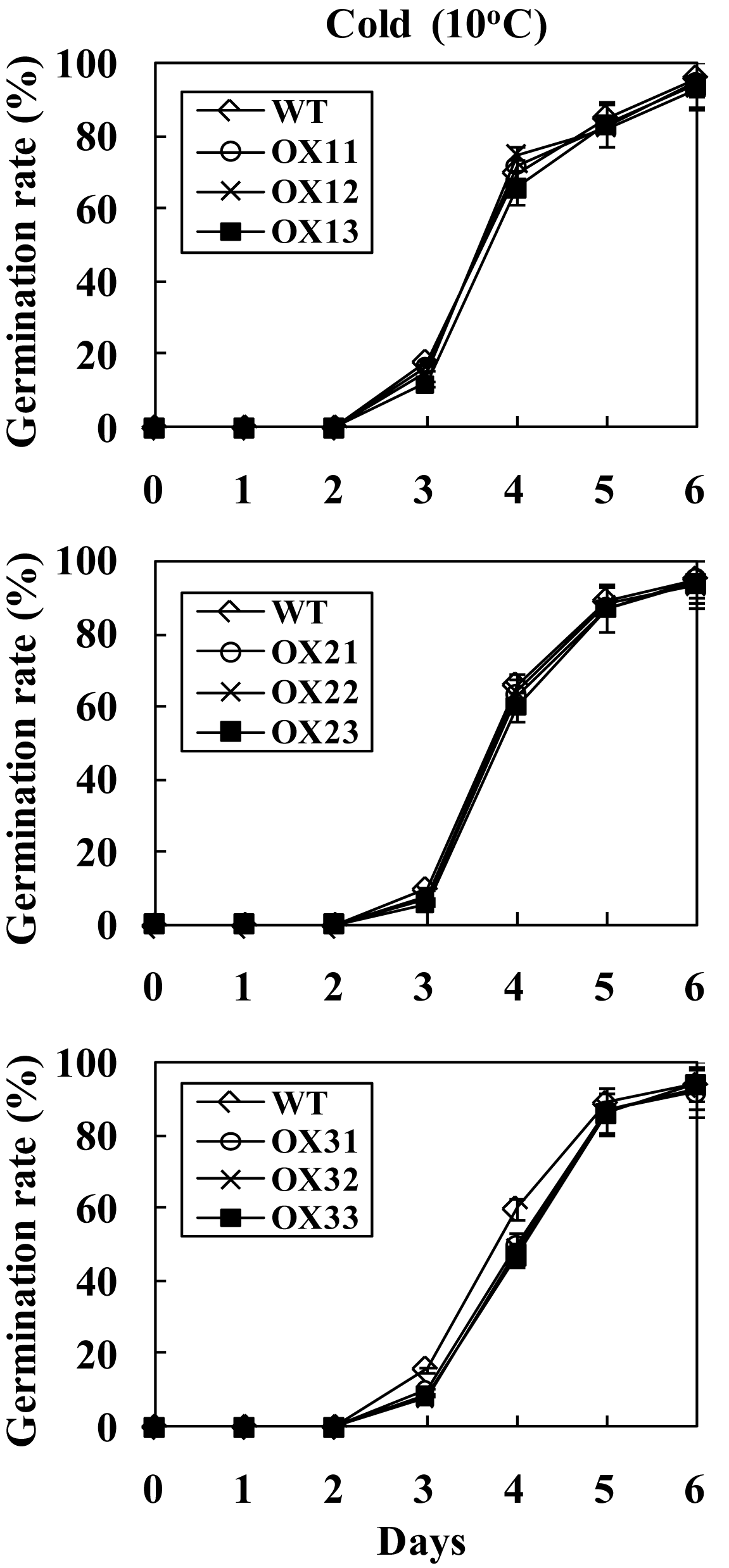

Supplement: Figure S3 — Seed germination of wild-type and transgenic plants under normal conditions. Seeds of wild type (Col-0) and overexpression plants 35S::TaRZ1 (OX11, OX12 and OX13) and 35S::TaRZ2 (OX21, OX22 and OX23) and 35S::TaRZ3 (OX31, OX32, and OX33) were germinated on MS medium at 24°C, and germination rates were scored at the indicated days. (TIF) [file pone.0096877.s003.tif]

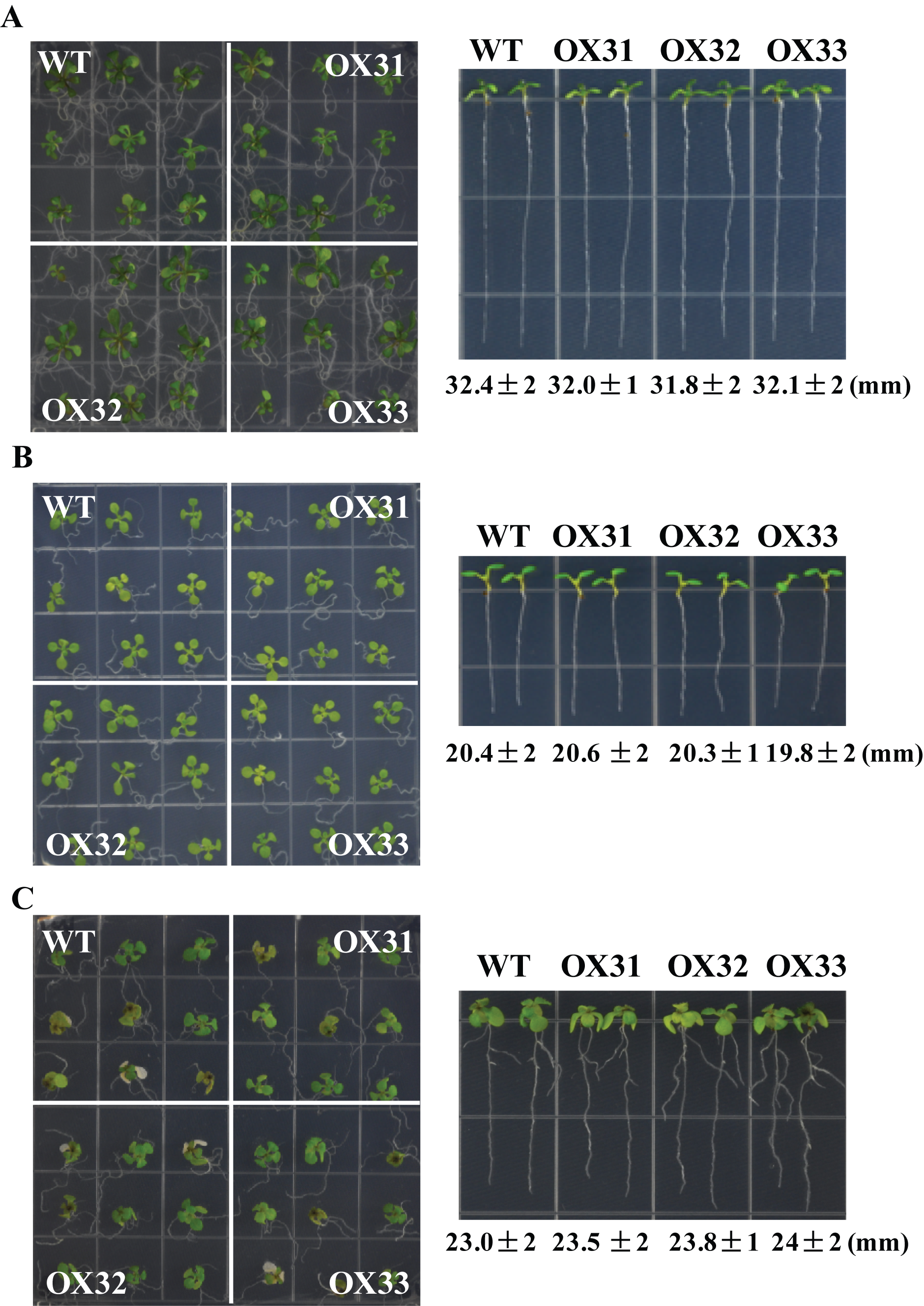

Supplement: Figure S4 — Effect of dehydration, cold and salt stress on seeding growth of wild type and 35S::TaRZ3 transgenic plants. (A) Effect of dehydration on seeding growth and root length of wild type and 35S::TaRZ3 transgenic plants. MS medium supplemented with 300 mM mannitol. (B) Effect of low temperature on seeding growth and root length of wild type and 35S::TaRZ3 transgenic plants. (C) Effect of salt on seeding growth and root length of wild type and 35S::TaRZ3 transgenic plants. MS medium was supplemented with 125 mM NaCl. Values are means ± SD of at least 20 seedlings. (TIF) [file pone.0096877.s004.tif]

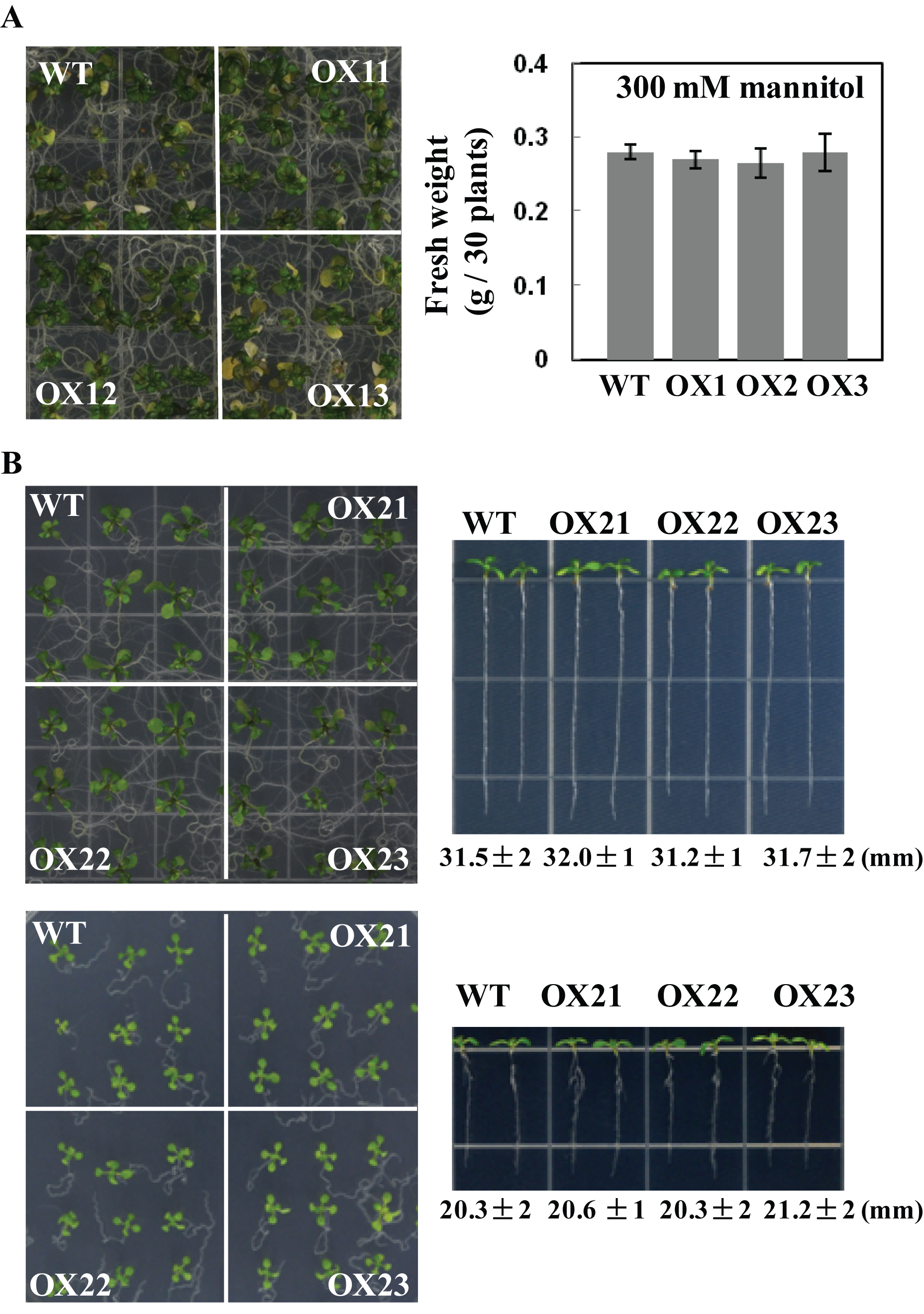

Supplement: Figure S5 — Effect of dehydration and cold stress on seeding growth of wild type and 35S::TaRZ1 transgenic plants and 35S::TaRZ2 transgenic plants. (A) Effect of dehydration on seeding growth and root length of wild type and 35S::TaRZ1 transgenic plants. (B) Effect of dehydration and low temperature on seeding growth and root length of wild type and 35S::TaRZ2 transgenic plants. Seedling and root growths of wild type (Col-0) and overexpression plants (OX21 OX22, and OX23) were examined on MS medium supplemented with 300 mM mannitol. Values are means ± SD of at least 30 seedlings. (TIF) [file pone.0096877.s005.tif]

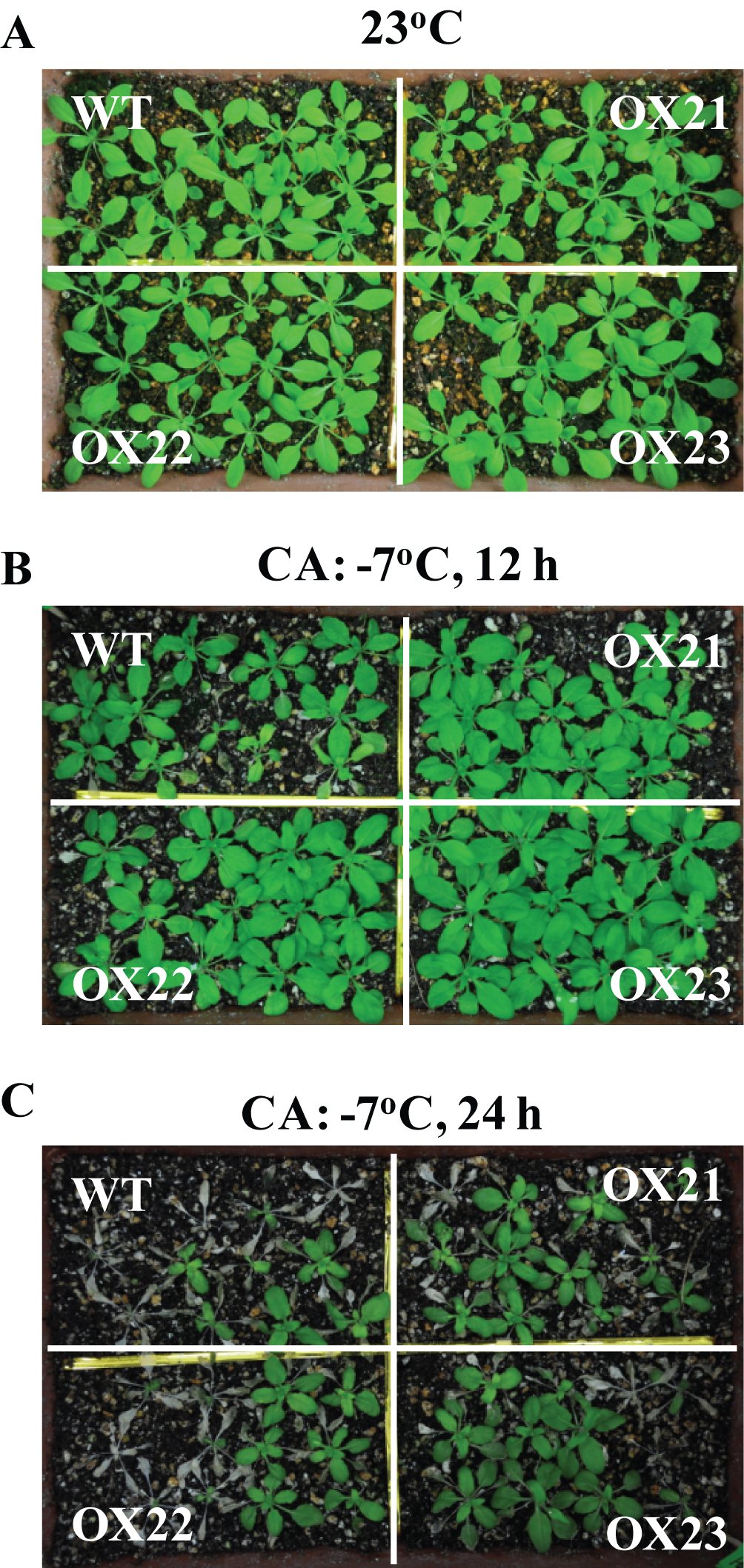

Supplement: Figure S6 — Cold-acclimated (CA) freezing tolerance test. Three-week-old plants were first placed at 4°C for 1 day, −1°C for 1 day, −7°C for 12–25 h, and then recovered under normal growth conditions for 7 d. The photograph shows a representative picture of repeated. (TIF) [file pone.0096877.s006.tif]
